# Following a Silent Metal Ion: A Combined X-ray Absorption and Nuclear Magnetic Resonance Spectroscopic Study of the Zn^2+^ Cation Dissipative Translocation between Two Different Ligands

**DOI:** 10.1021/acs.jpclett.2c01468

**Published:** 2022-06-13

**Authors:** Federico Frateloreto, Francesco Tavani, Marika Di Berto Mancini, Daniele Del Giudice, Giorgio Capocasa, Isabelle Kieffer, Osvaldo Lanzalunga, Stefano Di Stefano, Paola D’Angelo

**Affiliations:** † Dipartimento di Chimica, Università degli Studi di Roma “La Sapienza”, P.le A. Moro 5, I-00185 Rome, Italy; ‡ Observatoire des Sciences de l’Univers de Grenoble (OSUG), Université Grenoble-Alpes, UMR 832 CNRS, Grenoble, Cedex 9 F-38041, France; § BM30/CRG-FAME, ESRF, Polygone scientifique, Grenoble, 38000, France

## Abstract

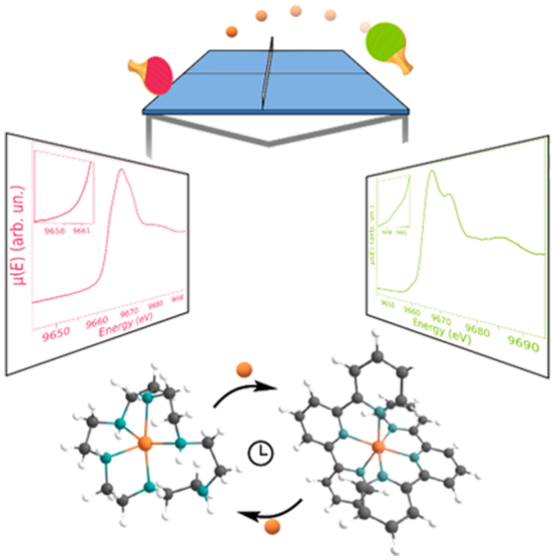

The dissipative translocation of the Zn^2+^ ion between two prototypical coordination complexes has been investigated by combining X-ray absorption and ^1^H NMR spectroscopy. An integrated experimental and theoretical approach, based on state-of-the-art Multivariate Curve Resolution and DFT based theoretical analyses, is presented as a means to understand the concentration time evolution of all relevant Zn and organic species in the investigated processes, and accurately characterize the solution structures of the key metal coordination complexes. Specifically, we investigate the dissipative translocation of the Zn^2+^ cation from hexaaza-18-crown-6 to two terpyridine moieties and back again to hexaaza-18-crown-6 using 2-cyano-2-phenylpropanoic acid and its *para*-chloro derivative as fuels. Our interdisciplinary approach has been proven to be a valuable tool to shed light on reactive systems containing metal ions that are silent to other spectroscopic methods. These combined experimental approaches will enable future applications to chemical and biological systems in a predictive manner.

Translocation, that is, the motion of a molecular entity (an ion or a neutral molecule) between two or more sites, is an ubiquitous process occurring in living systems. It is not a case that, recently, a great effort has been devoted to the realization of artificial translocators such as molecular walkers^1−8^ or exchange systems with precise functionalities (e.g., catalysis),^9−14^ able to mimic, at least in part, some features of biotic networks. Among these systems, we focused our attention on those operating under dissipative conditions,^15,16^ where the translocation of an ion or a neutral molecule **B**, from a site A to a site C (Figure 1a) persists as long as a fuel (a reactive species or radiative energy) is present. When the fuel is exhausted, a back-translocation restores the initial conditions, with **B** found again on site A. Some seminal papers from Schmittel’s group have recently appeared, which show how translocation under dissipative conditions allows the control over time of the solution fluorescence or of the efficiency of complex catalytic systems.^17−19^

**Figure 1:**
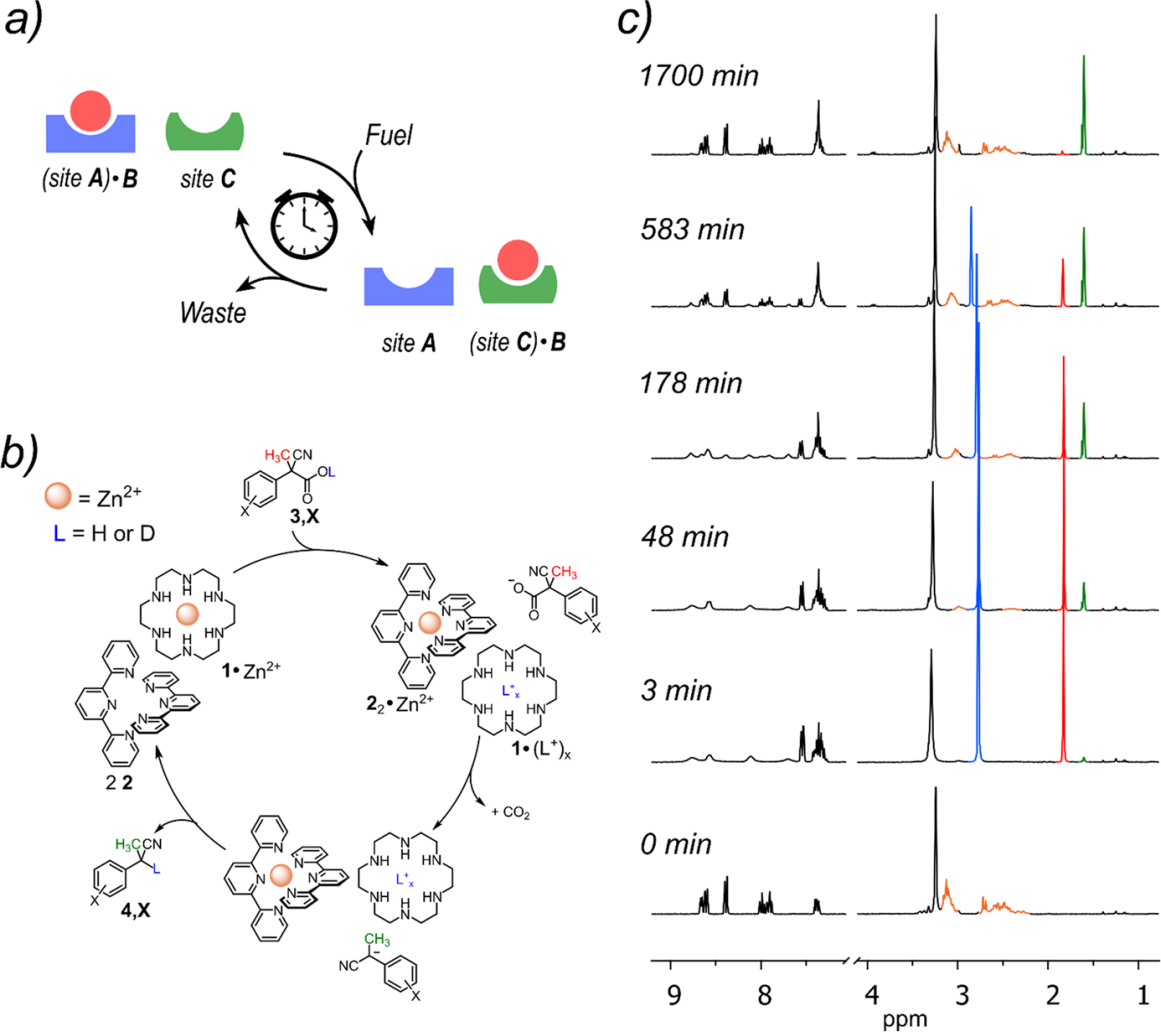
a) Dissipative translocation process: **B** passes from site A to site C and back again to site A under the action of a chemical fuel. (b) The Zn^2+^ ion is induced to abandon ligand **1** to form complex **2**_2_•Zn^2+^ by the addition of the fuel acid **3,X**. Eventually, consumption of the fuel to waste product **4,X** causes the restoration of complex **1**•Zn^2+^. (c) ^1^H NMR monitoring of a dissipative cycle **1**•Zn^2+^ → **2**_2_•Zn^2+^ → **1**•Zn^2+^ triggered by fuel acid **3,H** (see text for color code).

The Zn^2+^ cation is a diamagnetic ion with a filled 3d shell, and for this reason it is not easily investigated through spectroscopic techniques.^20^ For instance, UV–vis spectroscopy, magnetic circular dichroism, or electron paramagnetic resonance (EPR) are not effective in the study of the Zn^2+^ cation,^21^ while the use of ^67^Zn-NMR presents limitations such as low natural abundance and small magnetic moment of the ^67^Zn nucleus.^22^ Therefore, applications of these spectroscopic techniques are not well suited for following a chemical reaction or other kinetic changes involving the Zn^2+^ ion. X-ray absorption spectroscopy (XAS) provides element-specific structural and electronic information on the local structure around a photoabsorbing atom and is applicable to both solid and solution samples. Consequently, leveraging the insight provided by XAS to investigate Zn^2+^-based systems in solution may allow one to gain valuable and unrivaled information on the reactive, structural, and electronic properties of the Zn^2+^ species at play.^23−27^ Moreover, the combined use of different experimental techniques to monitor the advancement of a chemical process is often a powerful tool for a satisfactory understanding of the operating reaction mechanism. XAS has been successfully coupled with UV–vis to follow chemical reactions involving nickel, iridium, palladium, and iron complexes in solution.^28,29^ An alternative of this combined experimental approach is to couple XAS with ^1^H NMR as both techniques can be used to investigate reactive systems containing metal ions that are impossible to measure with other spectroscopic methods. This strategy has been successfully applied in a recent study to obtain mechanistic information on the reactive pathway of the Cu^2+^ ion that undergoes a ligand exchange process.^26^

Here, we report that the use of the XAS/^1^H NMR coupled technique enables an easy monitoring of the dissipative translocation of the Zn^2+^ cation from hexaaza-18-crown-6 (**1**, Figure 1b) to two terpyridine (**2**) moieties and back again to hexaaza-18-crown-6. As convenient fuels 2-cyano-2-phenylpropanoic acid (**3,H**)^30,31^ and its *para*-chloro derivative (**3,Cl**)^32−34^ have been employed.

In a first experiment monitored by ^1^H NMR, equimolar amounts (5.0 mM) of hexaaza-16-crown-8 (**1**) and Zn(OTf)_2_ were mixed in a CD_2_Cl_2_/CD_3_OD 9:1 solution at 25 °C. Under these conditions complex **1**•Zn^2+^ is quantitatively formed, giving rise to the orange signals in the bottom trace (0 min) reported in Figure 1c, which attest the nonequivalence of the methylene protons in the complex (*vide infra*). The black signals between 9 and 7 ppm (again see trace at 0 min) belong to terpyridine **2** (10.0 mM), also added to the solution, which is free and noninteracting with the Zn^2+^ cation. As stated before, the latter is instead intimately enclosed in the hexaazacrown ether ligand. At this point fuel **3,H** (20.0 mM) is added. The ^1^H NMR spectrum recorded just after the addition of the fuel (trace at 3 min, Figure 1c), clearly shows that the translocation of the Zn^2+^ cation from hexaaza18-crown-6 **1** to two residues of terpyridine has occurred. The transformation **1**•Zn^2+^ → **2**_2_•Zn^2+^ is indeed witnessed by the strong broadening of the terpyridine signals (from 9 to 7) and by the substitution of the complex orange pattern belonging to **1**•Zn^2+^ by the sharp blue singlet at 2.60 ppm due to the protonated **1**,^35^ which has lost affinity for the zinc cation. In other words, the fuel acid has protonated **1**, and, consequently, the Zn^2+^ cation has translocated into two terpyridine moieties to form complex **2**_2_•Zn^2+^. From now onward, the fuel is slowly consumed due to the decarboxylation of the carboxylate anion, **1** deprotonated again by the just formed carbanion (read clockwise the scheme in Figure 1b), and, at the end of the process, complex **1**•Zn^2+^ is definitely re-established as can be easily seen by comparing traces at 0 and 1700 min in Figure 1c (traces are superimposable apart from the signals due to the waste products). The contextual consuming of fuel acid **3,H** to waste product **4,H** can be followed by the decrease of the red singlet related to the methyl group of the former and the increase of the green signal related to the same group in the latter (Figure 1c). A similar experiment, has been carried with acid **3,Cl** instead of acid **3,H** (see SI). As expected,^32^ in this case, the translocation cycle was faster although featured by the same characteristics.

Subsequently, XAS spectra at the Zn K-edge were collected and analyzed to obtain structural and electronic insights into the Zn^2+^ complexes key to the investigated reactive processes, and to directly retrieve information on how their concentration evolves in the reaction mixtures. The Zn K-edge X-ray absorption near edge structure (XANES) spectra of the reference complexes **1**•Zn^2+^ and **2**_2_•Zn^2+^ were collected on CH_2_Cl_2_/MeOH 9:1 solutions of Zn(OTf)_2_ (5.0 mM) and (i) **1** (5.0 mM) or (ii) **2** (10.0 mM), and are shown in Figure 2 panels c and d, respectively.

**Figure 2:**
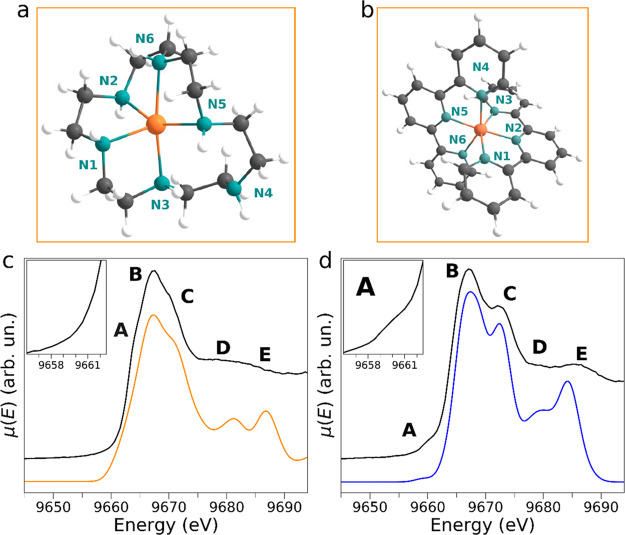
(a,b) Minimum energy structures calculated at the DFT/ZORA-def2-TZVP level for complexes **1**•Zn^2+^ (a) and **2**_2_•Zn^2+^ (b), where each nitrogen atom is labeled (N1–N6). The Zn^2+^ cation is depicted in orange, while the nitrogen, carbon, and hydrogen atoms are shown in green, black, and white, respectively. (c,d) Comparison between the experimental and theoretical Zn K-edge XANES spectra of **1**•Zn^2+^ (c) and **2**_2_•Zn^2+^ (d). The experimental curves are depicted with full black lines while the simulated ones belonging to the **1**•Zn^2+^ and **2**_2_•Zn^2+^ complexes are shown in yellow (c) and blue (d), respectively. Magnifications of the energy region associated with transition A (absent in the XAS spectrum of **1**•Zn^2+^ and present in that of **2**_2_•Zn^2+^) are shown in the insets.

The rise of intensity at the Zn K-edge is due to the absorption of an incoming photon and since the oxidation state of Zinc is 2+ in both complexes, the edge energies of the two XAS spectra do not differ significantly and are approximately both equal to 9664 eV.^20^ Conversely, the XANES spectra of **1**•Zn^2+^ and **2**_2_•Zn^2+^ exhibit clear differences in the relative intensities and positions of the features located at higher energy (features C, D, and E), due to the diverse structural arrangement around the photoabsorber. Note that the XAS spectrum of complex **2**_2_•Zn^2+^ has a distinctive shoulder at 9673.1 eV (feature C), which is less pronounced in the spectrum of complex **1**•Zn^2+^. Interesting experimental evidence is that the XAS spectrum of complex **2**_2_•Zn^2+^ possesses a small but clearly detectable pre-edge transition located at ca. 9659.9 eV (transition A, inset of Figure 2d) which is absent in the spectrum of **1**•Zn^2+^ (see inset of Figure 2c). It is well-known that pre-edge features predominantly occur because of a 1s to 3d transition, and should therefore not be theoretically possible in metals with filled 3d orbitals such as Zn^2+^.^20,21^ However, pre-edges have been observed in other nominally d^10^ metal complexes, such as Cu^+^ complexes,^36−38^ and explained as resulting from metal-to-ligand charge transfer (MLCT).^20,36−38^ Moreover, the presence of pre-edge features were observed in Zn^2+^ complexes with tripodal ligands and was attributed to a MLCT excitation into low lying pyridine π* orbitals.^39^ Finally, the experimental XAS spectrum of **1**•Zn^2+^ shows the presence of a shoulder at the rising edge (transition A, Figure 2c) that has been assigned to a dipole allowed 1s to 4p transition.^20^

In order to better understand the structural and electronic properties of the investigated Zn^2+^ species, time dependent DFT (TDDFT) theoretical spectra^50,51^ were calculated by means of the ORCA code^40^ starting from DFT optimized geometrical models of the **1**•Zn^2+^ and **2**_2_•Zn^2+^ complexes. The associated DFT optimized structures of **1**•Zn^2+^ and **2**_2_•Zn^2+^ are shown in Figure 2 panels a and b, respectively, while Table S1 lists the relevant structural parameters. The DFT optimized structure of **1**•Zn^2+^ is that of a distorted square pyramid, where the Zn^2+^ cation is coordinated by five of the six nitrogen atoms of the aza-crown species. In this geometry, the average equatorial Zn–N bond length is equal to 2.274 Å, while the distance between the Zn^2+^ cation and the axial nitrogen atom (N1, Figure 2a) is 2.145 Å. Conversely, the DFT optimized structure of **2**_2_•Zn^2+^ is that of a distorted octahedron, that may be described as the superposition of a “tetragonal compression” along the [001] direction due to the rigidity of the terpyridine ligands and a “tetragonal elongation” perpendicular to the [001] direction, similarly to the structure of the complex established between terpyridine and the Cu^2+^ cation (X-ray data^41^). As a result, the Zn–N average axial and equatorial bond lengths are equal to 2.095 and 2.194 Å, respectively. These results are in good agreement with the previously reported crystal structure of bis(2,2′:6′,2′′-terpyridine)zinc(II) dinitrate dihydrate, where the zinc atom is irregularly six-coordinated by the six N atoms from the two terpyridine ligands in the same distorted octahedral environment, and all Zn–N bond lengths are in the range of 2.084(4)–2.187(2) Å.^42^

The TDDFT theoretical Zn K-edge XAS spectra calculated for the optimized clusters of complexes **1**•Zn^2+^ and **2**_2_•Zn^2+^ are shown in Figure 2 panels c and d, respectively, and compared to the experimental curves. One may note that the general agreement between the experimental and TDDFT theoretical XAS spectra of both complexes is quite good, and that the XANES calculations reproduce both the energies and relative intensities of all relevant features present in **1**•Zn^2+^ and **2**_2_•Zn^2+^ (Figure 2c). Further, a pre-edge transition is present in the theoretical XAS spectrum of **2**_2_•Zn^2+^, while it is absent in that of **1**•Zn^2+^, in line with the experimental data. In general, the computations showed that the pre-edge feature for the **2**_2_•Zn^2+^ complex consists of two distinct transitions A_1_ and A_2_ resulting from core excitation into the molecular LUMO and LUMO+1, as shown in Figure 3, where the individual A_1_ and A_2_ transitions contributing to the pre-edge peak are plotted together with the associated acceptor orbitals. Conversely, the percentage element contributions to the HOMO, LUMO, and LUMO+1 of **2**_2_•Zn^2+^ are shown in Figure S4, while Figures S5 and S6 present, respectively, the percentage contribution of each atom and of the Zn relevant orbitals to the same three molecular orbitals of **2**_2_•Zn^2+^. According to the DFT analysis, the LUMO and LUMO+1 of **2**_2_•Zn^2+^ are predominantly of ligand π* character. The metal in fact contributes solely 1.1% d_*xz*_ and 0.8% d_*yz*_ character to the two orbitals (Figure S6), respectively, with a small p admixture (<1%), and the total Zn contribution to the LUMO and LUMO+1 does not exceed ca. 2% (Figure S5). These findings lend support to the assignment of the pre-edge feature of **2**_2_•Zn^2+^ as a MLCT band.^20,36−38^

**Figure 3:**
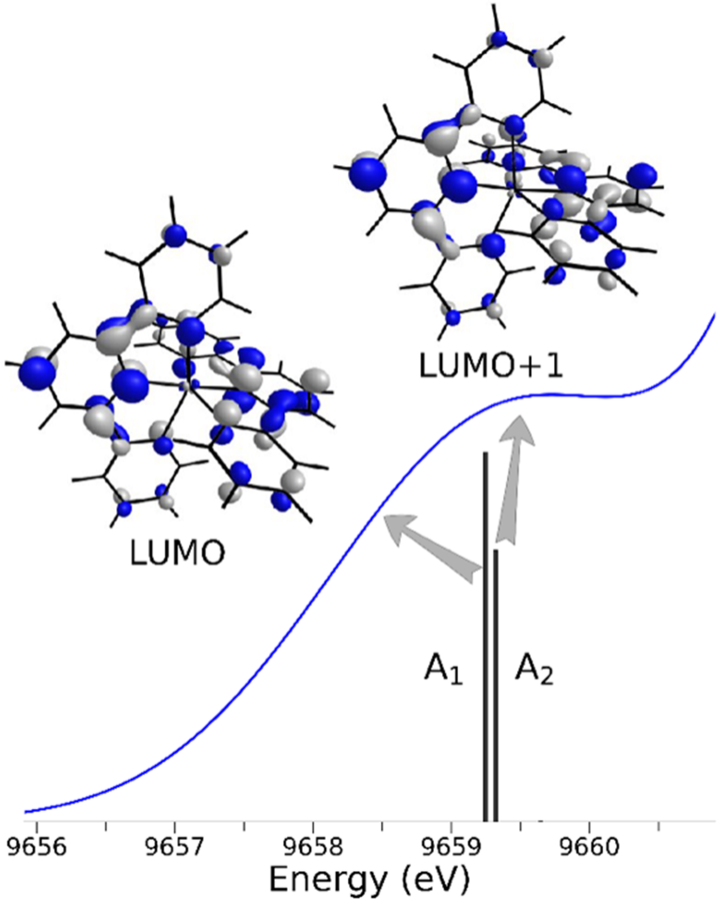
Enlargement of the pre-edge transition region of the theoretical TDDFT XANES spectrum of complex **2**_2_•Zn^2+^ (blue curve). The two individual contributions (A_1_ and A_2_) to the simulated curve are evidenced by black vertical bars, and the acceptor orbitals (the LUMO and LUMO+1) are drawn.

Having elucidated the structural properties of complexes **1**•Zn^2+^ and **2**_2_•Zn^2+^ and having proposed the assignment of the pre-edge transition present in the XAS spectrum of **2**_2_•Zn^2+^ as due to a MLCT, we may discuss the results concerning the use of XAS to investigate the reactive translocation process depicted in Figure 1b.

The Zn K-edge XANES spectra recorded for the reactions involving **1** (5.0 mM), Zn(OTf)_2_ (5.0 mM), **2** (10.0 mM), and (i) **3,H** (20.0 mM) or (ii) **3,Cl** (40.0 mM) (in both cases the reactants were mixed in CD_2_Cl_2_/CD_3_OD 9:1, at 25 °C) are shown in Figure 4a,b (i) and 4c,d (ii). Specifically, Figure 4 panels a and c render the XANES data of the two reactions in 3D plots, while Figure4 panels b and d present the same XAS data in 2D, where the first and last experimental spectra are evidenced by full red and blue lines, respectively.

**Figure 4:**
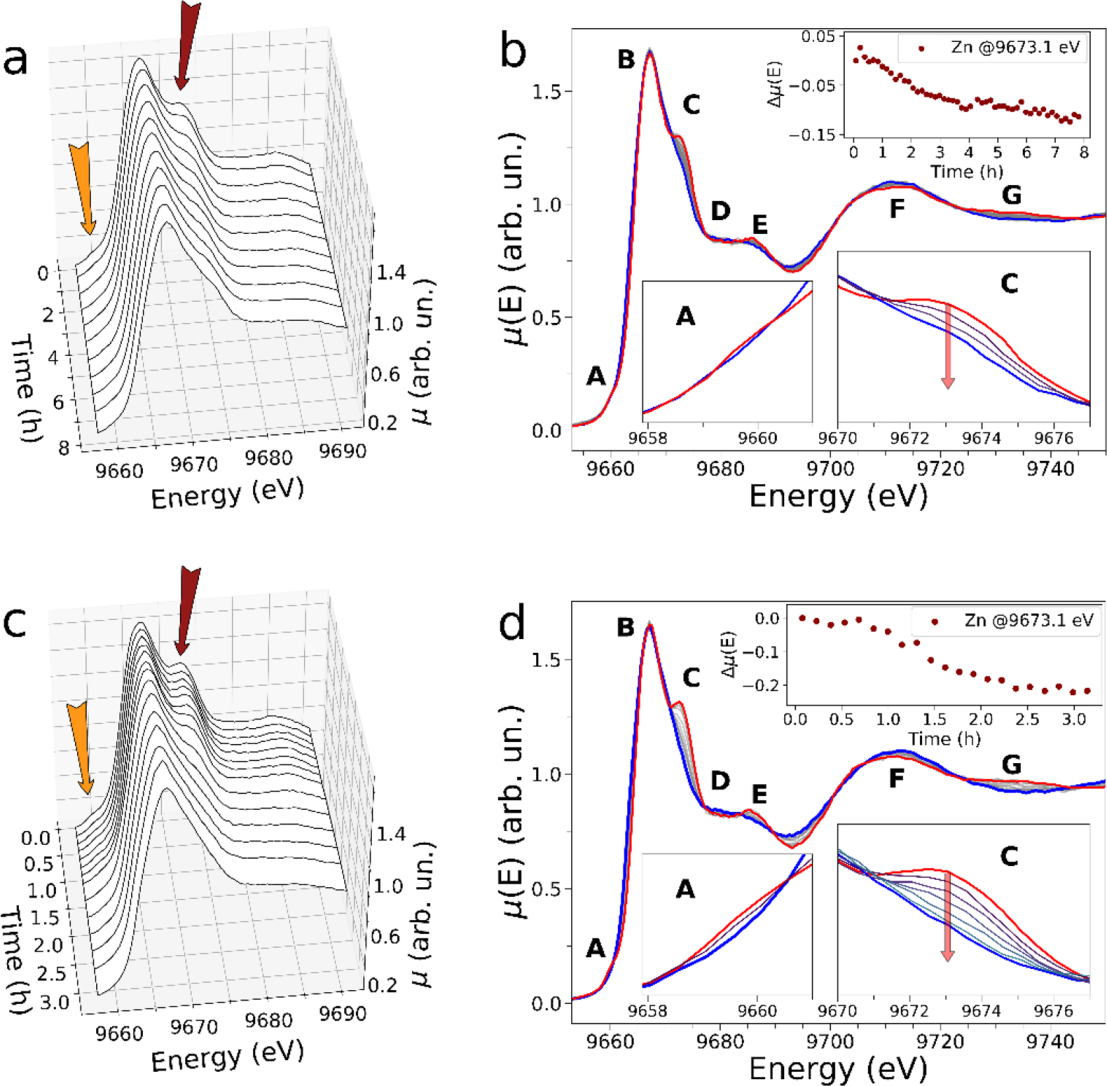
Time evolution of the Zn K-edge XANES spectra of the reactions involving **1** (5.0 mM), Zn(OTf)_2_ (5.0 mM), **2** (10.0 mM), and **3,H** (20.0 mM) (panels a,b) or **3,Cl** (40.0 mM) (panels c,d). In the 3D XANES data plots (panels a,c), orange and dark red arrows highlight the transitions located at 9659.9 and 9673.1 eV, respectively. In the 2D XANES data plots (panels b, d), the first and last XAS spectra are shown in red and blue, respectively, while the other reaction XAS spectra are depicted in gray. Here, the main features of the experimental XANES spectra are labeled with uppercase letters (A → G), while magnifications of the A and C transition regions, as well as the time evolution of the intensity difference measured at 9673.1 eV are shown in the insets.

As for the reaction involving fuel **3,H**, one may observe that the first recorded XAS spectrum (Figure 4b, red line) bears close resemblance with the XAS spectrum of complex **2**_2_•Zn^2+^ (see Figure 2c), although its features A and C appear to be slightly less intense than in the XAS spectrum of the terpyridine-based standard (see lower left and right insets of Figure 4b). As the reaction proceeds, the intensity measured in proximity of feature C at 9673.1 eV decreases as shown in the upper inset of Figure 4b, the pre-edge transition is largely depleted, and the last recorded XANES spectrum (full blue line) exhibits strong similarities with that of **1**•Zn^2+^. This evidence supports the notion that once the first XAS spectrum has been collected (0.0 ≤ *t* ≤ 9.2 min from reaction start) the Zn^2+^ cation has already translocated from **1** to **2** a first time, and the second **2**_2_•Zn^2+^ → **1**•Zn^2+^ transformation has started to occur due to the decarboxylation of the fuel.

The latter process is relatively slow, and its duration exceeds 7 h. On the contrary, looking at Figure 4c,d it appears that the reaction involving fuel **3,Cl** is significantly faster, with clear spectral changes evident in the data collected in the first 3 h from the reaction start. Note that the first XAS spectrum collected during the reaction (Figure 4d, red line) exhibits more pronounced A and C features (see Figure 4c and insets of Figure 4d) if compared to those of the first measured XAS spectrum of the reaction involving fuel **3,H** (see Figure 4a and insets of Figure 4b). Further, the intensity measured at 9673.1 eV (upper inset of Figure 4d) remains approximately stationary up to *t* ∼ 0.6 h from reaction start and then begins to decay. In fact, the excess of fuel added to the reaction mixture (40.0 mM of **3,Cl**) allows complex **2**_2_•Zn^2+^, formed after the “fast” initial translocation of the Zn^2+^ cation from **1** to **2**, to be preserved for a longer time interval and to be evidenced by the XAS measurements before its decay, once the excess of **3,Cl** is consumed. Also in this case, the last reaction XAS spectrum possesses largely depleted A and C transitions and bears strong similarities with that of the **1**•Zn^2+^ standard.

To obtain information on the concentration time evolution of the **1**•Zn^2+^ and **2**_2_•Zn^2+^ complexes during the investigated reactive processes, a multivariate curve resolution (MCR) analysis^43−49^ was applied to both experimental XANES data sets employing a number of components equal to 2 (please refer to the Supporting Information for a more detailed description of the implemented method). Owing to the Lambert–Beer law, one may in fact view each experimental XANES spectrum as stemming from the concentration weighed contribution of the reference XANES spectra of **1**•Zn^2+^ and **2**_2_•Zn^2+^. In the decomposition, the two spectral components were constrained to coincide with the XANES spectra of **1**•Zn^2+^ and **2**_2_•Zn^2+^ and are shown in Figure 5a. Conversely, Figure 5 panels b and c show the associated MCR concentration profiles (dotted lines) relative to the translocation reactions involving fuels **3,H** and **3,Cl**, respectively.

**Figure 5:**
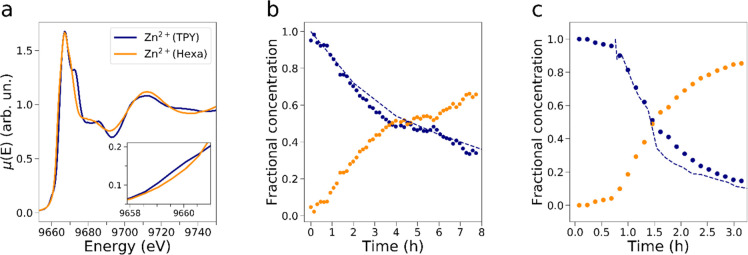
Results of the MCR decomposition applied to the collected Zn K-edge XANES spectra for the investigated reactions. The XANES extracted spectra assigned to the reaction key species **1**•Zn^2+^ (orange line) and **2**_2_•Zn^2+^ (blue line) (a) and time evolution of the associated fractional concentration profiles for the reactions involving fuels **3,H** (b) and **3,Cl** (c) are shown. The concentration time evolution of complex **2**_2_•Zn^2+^ (once the excess acid has been consumed) evaluated from ^1^H NMR data is also presented in panels b and c (dashed blue lines).

As expected, in the reaction involving fuel **3**,**H** the fractional concentration of **2**_2_•Zn^2+^ decreases over time while that of **1**•Zn^2+^ increases, with the relative abundance of **2**_2_•Zn^2+^ starting from a value of ca. 95% and reaching one of ca. 34% at *t* ∼ 7.7 h from reaction start. Notably, the time evolution of the concentration of **2**_2_•Zn^2+^ derived by the MCR analysis (Figure 5b, dotted blue line) is in excellent agreement with that estimated from the ^1^H NMR reaction data for the same complex (Figure 5b, dashed blue line). Conversely, in the process involving fuel **3,Cl** the fractional concentration of **2**_2_•Zn^2+^ starts from ca. 100% and, after an initial quasi-stationary period during which the excess fuel is still being consumed, starts to readily decrease at *t* ∼ 0.6 h. According to the MCR analysis, complexes **2**_2_•Zn^2+^ and **1**•Zn^2+^ reach percentage concentration values of ca. 15% and 85% at *t* ∼ 3.1 h. Also in this case the ^1^H NMR derived concentration evolution of **2**_2_•Zn^2+^ (Figure 5c, dashed blue line) is in excellent agreement with that extracted from the MCR decomposition of the XAS data (Figure 5c, dotted blue line).

In conclusion, the combined NMR-XAS analysis has been found to be a very effective tool to provide detailed mechanistic and structural insights into the dissipative translocation process occurring between two prototypical Zn^2+^ coordination complexes.

This innovative experimental approach combines the sensitivity of the XAS technique to the metal ion close environment, with the capability of the ^1^H NMR spectroscopy to disclose the structure of the organic portion of metal complexes, thus allowing a thorough, microscopic characterization of the intermediate species formed during the reaction. In particular, the two experimental techniques allowed us to disclose different aspects of the reactive processes. On the one hand, XAS has been used to rationalize in detail how the local structural and electronic environment of the Zn^2+^ site evolves when the metal ion is transferred from the distorted square pyramid complex **1**•Zn^2+^ to the distorted octahedral complex **2**_2_•Zn^2+^. It is important to underline that such information is neither obtainable by ^1^H NMR alone, nor easily obtained by other spectroscopic methods due to the previously discussed experimental limitations involved in the investigation of the Zn^2+^ cation. On the other hand, the use the ^1^H NMR technique allowed us to track the evolution of the reaction organic components that is not detectable by the XAS spectroscopy. Remarkably, this combined approach can be used to study reactive processes involving spectroscopically quiet metals that can be hardly investigated by other experimental spectroscopic methods.

Leveraging our innovative experimental and theoretical approach, based upon TDDFT and MCR analyses, we determined the concentration time evolution of all relevant Zn and organic species in the investigated processes, accurately characterized the solution structures of the key metal coordination complexes, and provided evidence of a pre-edge XAS transition occurring in a Zn^2+^ based complex, although Zn^2+^ is formally a d^10^ cation. Our results disclose some intriguing aspects of the rich solution chemistry involving the Zn^2+^ cation and pave the way for a wider application of this combined approach to the study of chemical and biological processes involving spectroscopically quiet metal ions.

## Supporting Information Available

Additional experimental NMR spectra and details on the theoretical ORCA calculations and Multivariate Curve Resolution (MCR) procedure and results (PDF)
