# Supplementary material for: Following a Silent Metal Ion: A Combined X-ray Absorption and Nuclear Magnetic Resonance Spectroscopic Study of the Zn2+ Cation Dissipative Translocation between Two Different Ligands
Source: J Phys Chem Lett. 2022 Jun 13;13(24):5522–9. doi: 10.1021/acs.jpclett.2c01468 (PMC9234980; doi:10.1021/acs.jpclett.2c01468)
Supplement: Supplementary file 1 — jz2c01468_si_001.pdf [file jz2c01468_si_001.pdf]

Supporting Information

for

Following a Silent Metal Ion: A Combined X-Ray Absorption and Nuclear Magnetic Resonance Spectroscopic Study of the Zn^{2+} Cation Dissipative Translocation Between Two Different Ligands

Federico Frateloreto [a],§, *Francesco Tavani* [a],§, *Marika Di Berto Mancini* [a], *Daniele Del Giudice* [a], *Giorgio Capocasa*,[a] *Isabelle Kieffer* [b,c], *Osvaldo Lanzalunga* [a],*, *Stefano Di Stefano* [a],* *Paola D'Angelo* [a],*

[a] Dipartimento di Chimica, Università degli Studi di Roma "La Sapienza", P.le A. Moro 5, I-00185 Rome, Italy.

[b] Observatoire des Sciences de l'Univers de Grenoble (OSUG), Université Grenoble-Alpes, UMR 832 CNRS, Grenoble Cedex 9, F-38041, France

[c] BM30/CRG-FAME, ESRF, Polygone scientifique, Grenoble, 38000, France

E-mail: p.dangelo@uniroma1.it, stefano.distefano@uniroma1.it, osvaldo.lanzalunga@uniroma1.it

[§]Equal contribution.

Index

1. Experimental

1.1 Comparison between ^{1}H NMR spectra of 5.0 mM **1**, 5.0mM **1**•L^+, and 5.0mM **1**•Zn^{2+}.

1.2 ^{1}H NMR monitoring of the reaction between 20.0 mM **3**,**H** and 5.0 mM **1**•Zn^{2+} in the presence of 10.0 mM **2**.

1.3 ^{1}H NMR monitoring of the reaction between 20.0 mM **3**,**Cl** and 5.0 mM **1**•Zn^{2+} in the presence of 10.0 mM **2**.

1.4 X-ray absorption spectroscopy measurements.

2. Data Analysis

2.1 Theoretical Method

2.2 Structural parameters of the DFT optimized structures

2.3 Element, atomic and Zn^{2+} orbital contributions to the HOMO, LUMO and LUMO+1 of complex $\mathbf{2}_2$•Zn^{2+}

2.4 Decomposition of the XANES data into the spectra and fractional concentrations of key components

1. Experimental

^{1}H NMR measurements: ^{1}H NMR spectra were recorded on a 300 MHz spectrometer. The spectra were internally referenced to the residual proton signal of the solvent at 5.32 ppm (CD_2Cl_2). All the experiments were carried out at constant temperature (25 ˚C), using a mixture of CD_2Cl_2 / MeOD 9:1 as the solvent. General procedure for the reactions consisted in weighing 1.09 mg of $Zn(OTf)_2$ (3 μmol) into the NMR tube, which were dissolved in 300 μl of the above mentioned solvent. Then, 100 μl of a 30.0 mM stock solution of **1** (3 μmol) and 150 μl of a 40.0 mM stock solution of **2** (6 μmol) were added to the same tube, and the first spectrum was recorded (t=0 min). Finally, 50 μl of a 240 mM stock solution of **3,X** (**X** = **H** or **Cl**, 12 μmol) were added and the reaction was monitored until its completion. An analogue procedure has been used for XAS measurements using non-deuterated solvents.

1.1 Comparison between ^{1}H NMR spectra of 5.0 mM 1, 5.0mM 1•L$^+$, and 5.0mM 1•Zn^{2+}.

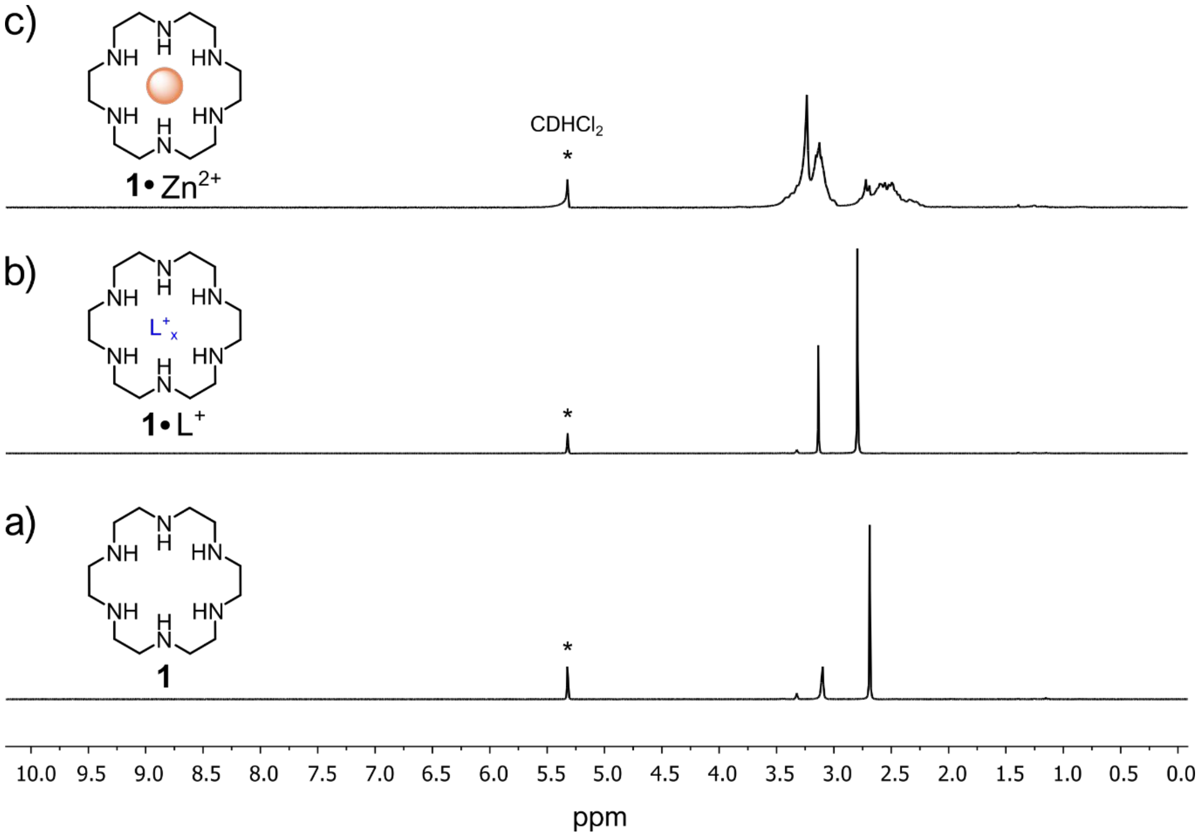

Figure S1. a) ^{1}H NMR spectrum (CD_2Cl_2 / MeOD 9:1 at 25 °C) of 5.0 mM **1** (trace a), 5.0 mM **1•L$^+$** (trace b), and 5.0mM **1•**Zn^{2+} (trace c).

1.2 ^{1}H NMR monitoring of the reaction between 20.0 mM 3,H and 5.0 mM 1•Zn^{2+} in the presence of 10.0 mM 2.

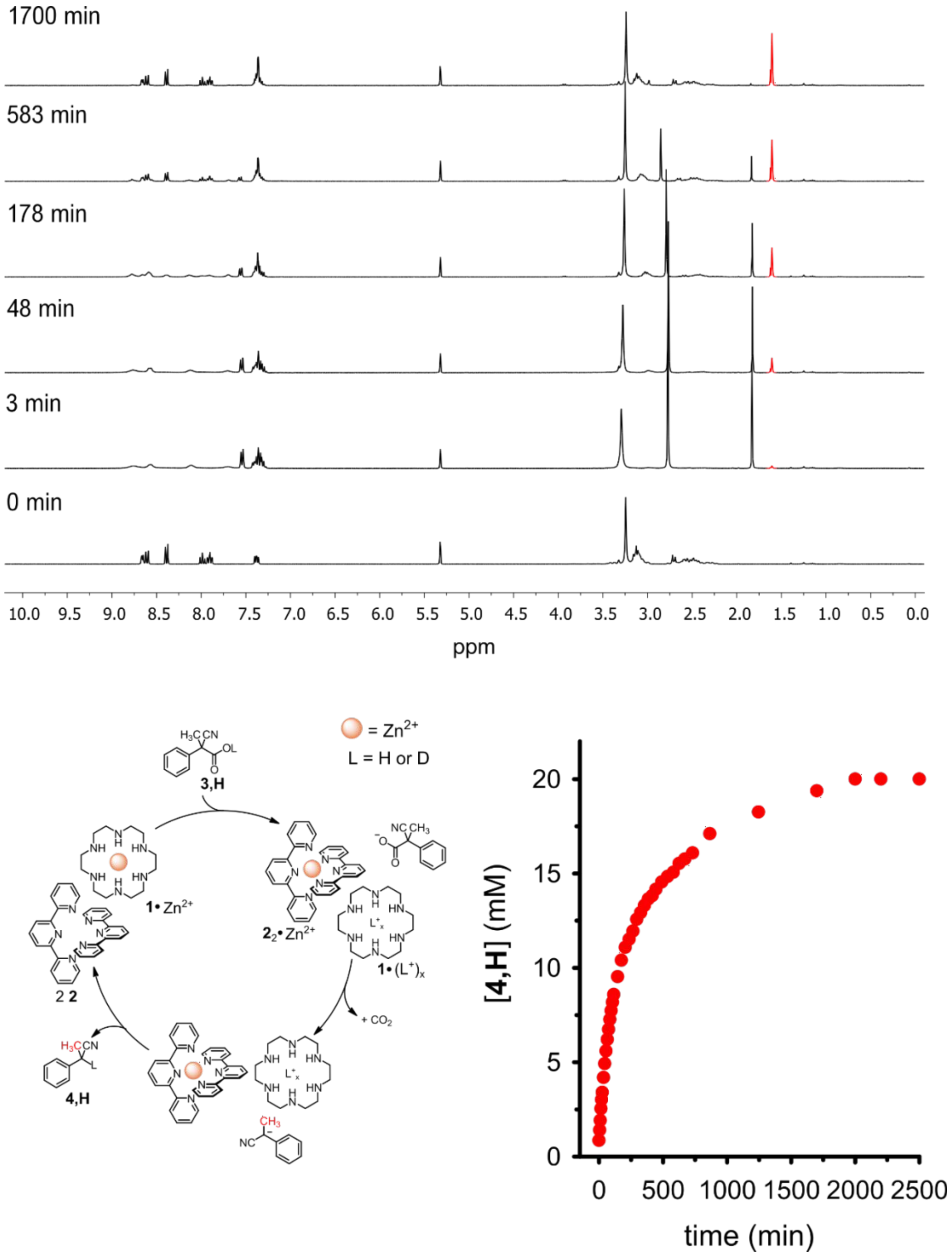

Figure S2. Top) ^{1}H NMR monitoring (CD_2Cl_2 / MeOD 9:1 at 25 ˚C) of 5.0 mM **1**•Zn^{2+} and 10.0 mM **2** solution, before (trace a) and after (traces b-f) the addition of 20.0 mM **3,H**, at the given reaction times. Bottom) Proposed reaction cycle (left), and time evolution of the reaction (right), obtained by

the integration of the signal related to the methyl group of the waste product **4,H** (1.60 ppm, red signal) over time.

1.3 ^{1}H NMR monitoring of the reaction between 20.0 mM 3,Cl and 5.0 mM 1•Zn^{2+} in the presence of 10.0 mM 2.

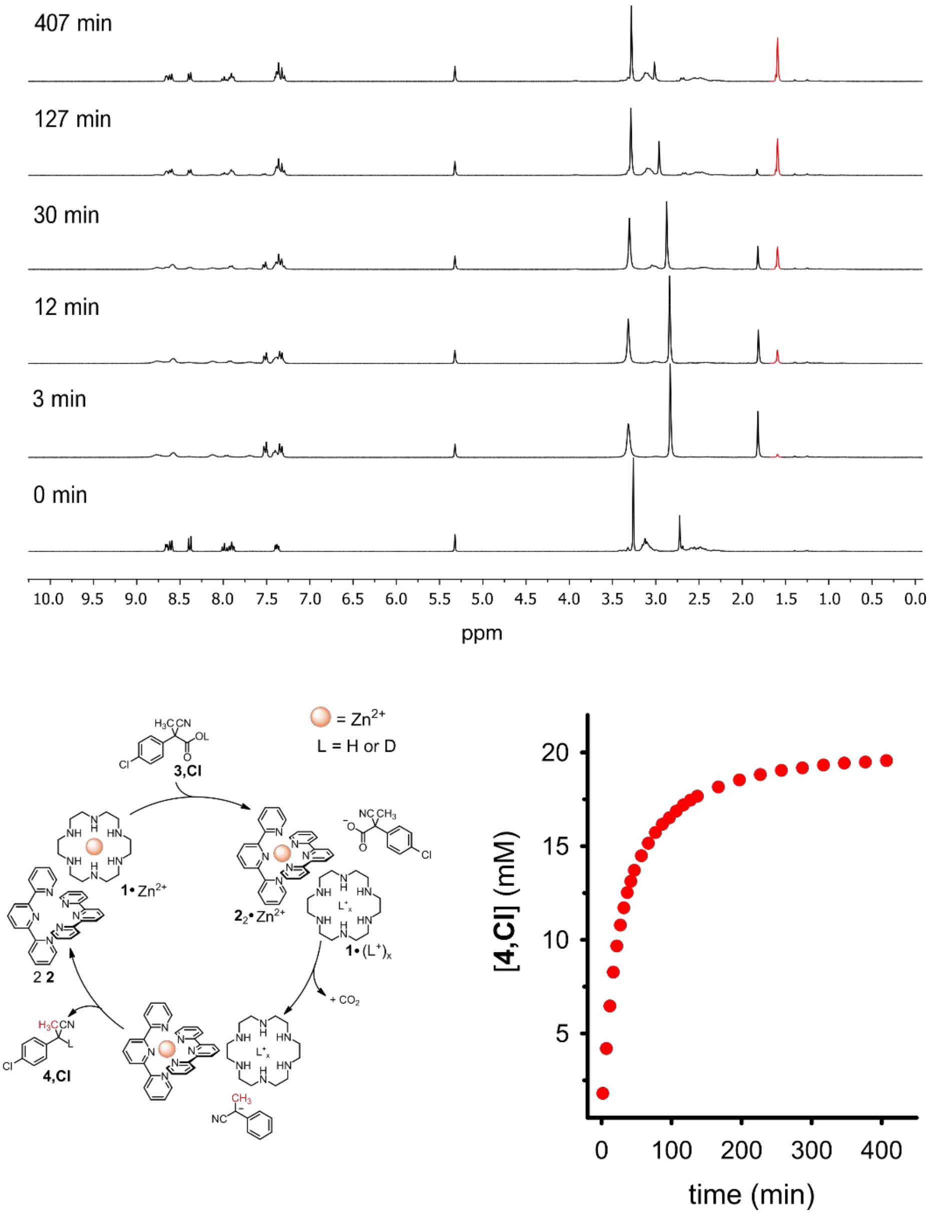

Figure S3. Top) ^{1}H NMR monitoring (CD_2Cl_2 / MeOD 9:1 at 25 ˚C) of 5.0 mM **1**•Zn^{2+} and 10.0 mM **2** solution, before (trace a) and after (traces b-f) the addition of 20.0 mM **3,Cl**, at the given reaction times. Bottom) Proposed reaction cycle (left), and time evolution of the reaction (right), obtained by the integration of the signal related to the methyl group of the waste product **4,Cl** (1.59 ppm, red signal) over time.

1.4 X-ray absorption spectroscopy measurements

X-ray absorption spectroscopy (XAS) measurements were carried out at the Zn K-edge at the BM30 beamline operating at the European Synchrotron Radiation Facility (ESRF). The beam was focused into a ca. 100 × 250 μm^2 (V × H, FWHM) spot on the sample, the monochromator was a DCM with Si(220) crystals and the photon flux was approximately 3×10^{11} photons s^{-1} (200 mA ring current). The energy calibration was carried out with a Zinc metallic foil by setting the first maximum of the derivative to 9.659 keV. Spectra were registered in fluorescence mode using a 13-element solid-state Ge detector. All X-ray Absorption Near Edge Structure (XANES) spectra were collected at 25 °C.

The XANES spectra of the **1**•Zn^{2+} and $\mathbf{2}_2$•Zn^{2+} reference compounds were collected on CH_2Cl_2/MeOH 9:1 solutions of $Zn(OTf)_2$ (5.0 mM) and (i) **1** (5.0 mM) or (ii) **2** (10.0 mM), respectively. As reported in main text of the manuscript, XANES spectra were recorded during the reactions of **1** (5.0 mM), $Zn(OTf)_2$ (5.0 mM), **2** (10.0 mM) and (i) **3**,H (20.0 mM) or (ii) **3**,Cl (40.0 mM). In both experiments, the reactants were mixed in CH_2Cl_2/MeOH 9:1.

2. Data analysis

2.1 Theoretical Method

Theoretical calculations were performed employing the ORCA 4.2.0 code.[1]

Geometries of complexes **1**•Zn^{2+} and $\mathbf{2}_2$•Zn^{2+} were optimized at the Density Functional Theory (DFT) theory level employing the B3LYP functional,[2-4] the D3BJ dispersion correction,[5] with a ZORA-def2-TZVP basis set.[6, 7] TDDFT theoretical spectra were aligned to the experimental spectra by applying a shift of –17.1 eV, a value determined as the average shift between the whitelines of the theoretical and experimental XAS spectra. The calculated TDDFT spectra were differentially broadened by a factor of 0.03*(Energy)^2, as previously described.[1]

2.2 Structural parameters of the DFT optimized structures

**1**•Zn^{2+}		**2**$_2$•Zn^{2+}	
Structural parameters	Theoretical values	Structural parameters	Theoretical values
d(Zn^{2+}-N1)	2.145 Å	d(Zn^{2+}-N1)	2.187 Å
d(Zn^{2+}-N2)	2.276 Å	d(Zn^{2+}-N2)	2.096 Å
d(Zn^{2+}-N3)	2.216 Å	d(Zn^{2+}-N3)	2.205 Å
d(Zn^{2+}-N4)	3.656 Å	d(Zn^{2+}-N4)	2.194 Å
d(Zn^{2+}-N5)	2.331 Å	d(Zn^{2+}-N5)	2.095 Å
d(Zn^{2+}-N6)	2.274 Å	d(Zn^{2+}-N6)	2.190 Å
a(N1- Zn^{2+}-N2)	81.8°	a(N1- Zn^{2+}-N2)	75.7°
a(N1- Zn^{2+}-N3)	80.6°	a(N1- Zn^{2+}-N3)	151.1°
a(N1- Zn^{2+}-N5)	118.6°	a(N1- Zn^{2+}-N4)	98.6°
a(N1- Zn^{2+}-N6)	108.7°	a(N1- Zn^{2+}-N5)	109.3°
a(N2- Zn^{2+}-N3)	106.1°	a(N1- Zn^{2+}-N6)	88.6°
a(N2- Zn^{2+}-N5)	152.5°	a(N2- Zn^{2+}-N3)	75.4°
a(N2- Zn^{2+}-N6)	74.9°	a(N2- Zn^{2+}-N4)	103.5°
a(N3- Zn^{2+}-N5)	95.9°	a(N2-Zn^{2+}-N5)	175.0°
a(N3- Zn^{2+}-N6)	170.7°	a(N2-Zn^{2+}-N6)	105.4°
a(N5- Zn^{2+}-N6)	80.8°	a(N3-Zn^{2+}-N4)	89.2°
-	-	a(N3-Zn^{2+}-N5)	99.6°
-	-	a(N3-Zn^{2+}-N6)	97.9°
-	-	a(N4-Zn^{2+}-N5)	75.6°
-	-	a(N4-Zn^{2+}-N6)	151.1°
-	-	a(N5-Zn^{2+}-N6)	75.6°

Table S1. Main structural parameters (bond lengths and relevant angles) of the DFT optimized structures of complexes **1**•Zn^{2+} (left side of the table) and **2**$_2$•Zn^{2+} (right side of the table). The listed bond lengths and angles are enclosed by parentheses and preceded by *d* and *a*, respectively.

2.3 Element, atomic and Zn^{2+} orbital contributions to the HOMO, LUMO and LUMO+1 of complex **2_2•Zn^{2+}**

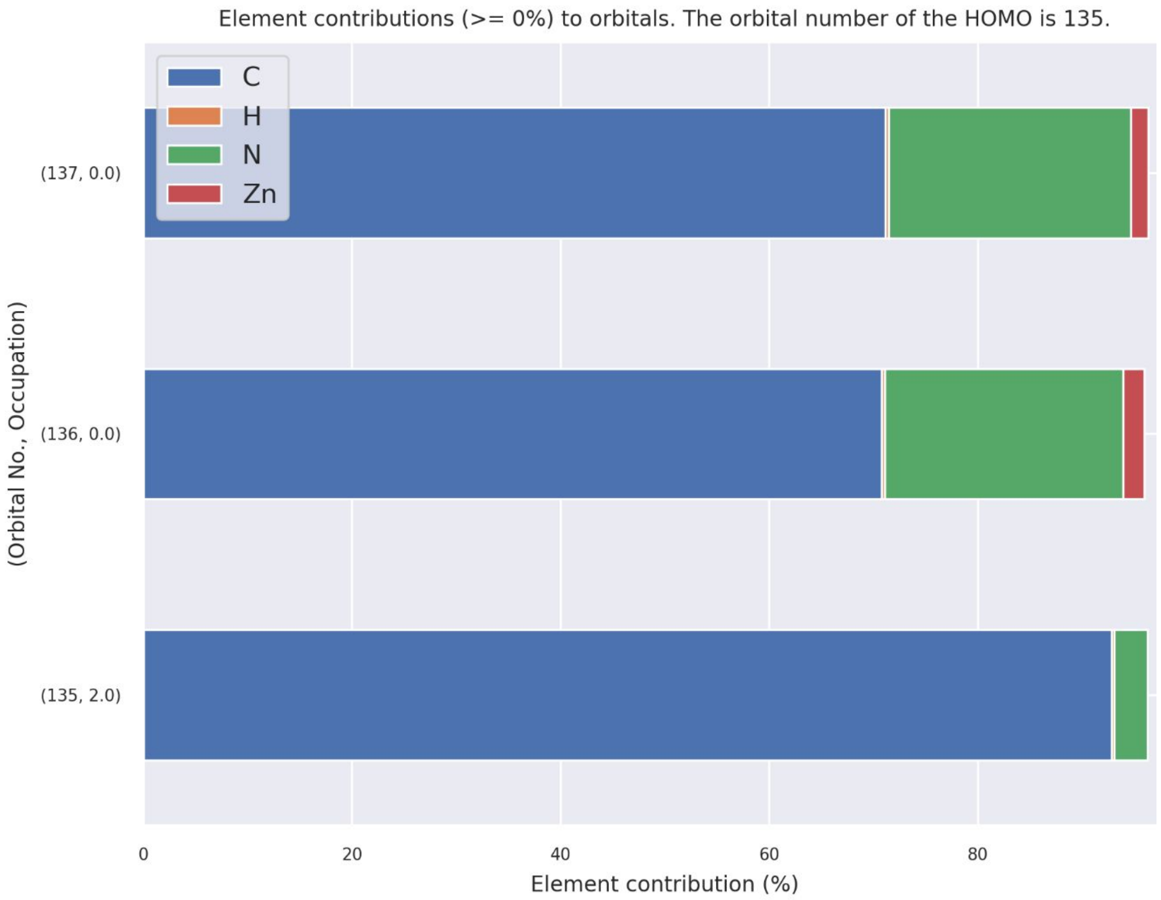

Figure S4. Element contribution (%) of Zn, N, C and H to the HOMO (orbital no. 135), LUMO (orbital no. 136) and LUMO+1 (orbital no. 137) of complex **2_2•**Zn^{2+}.

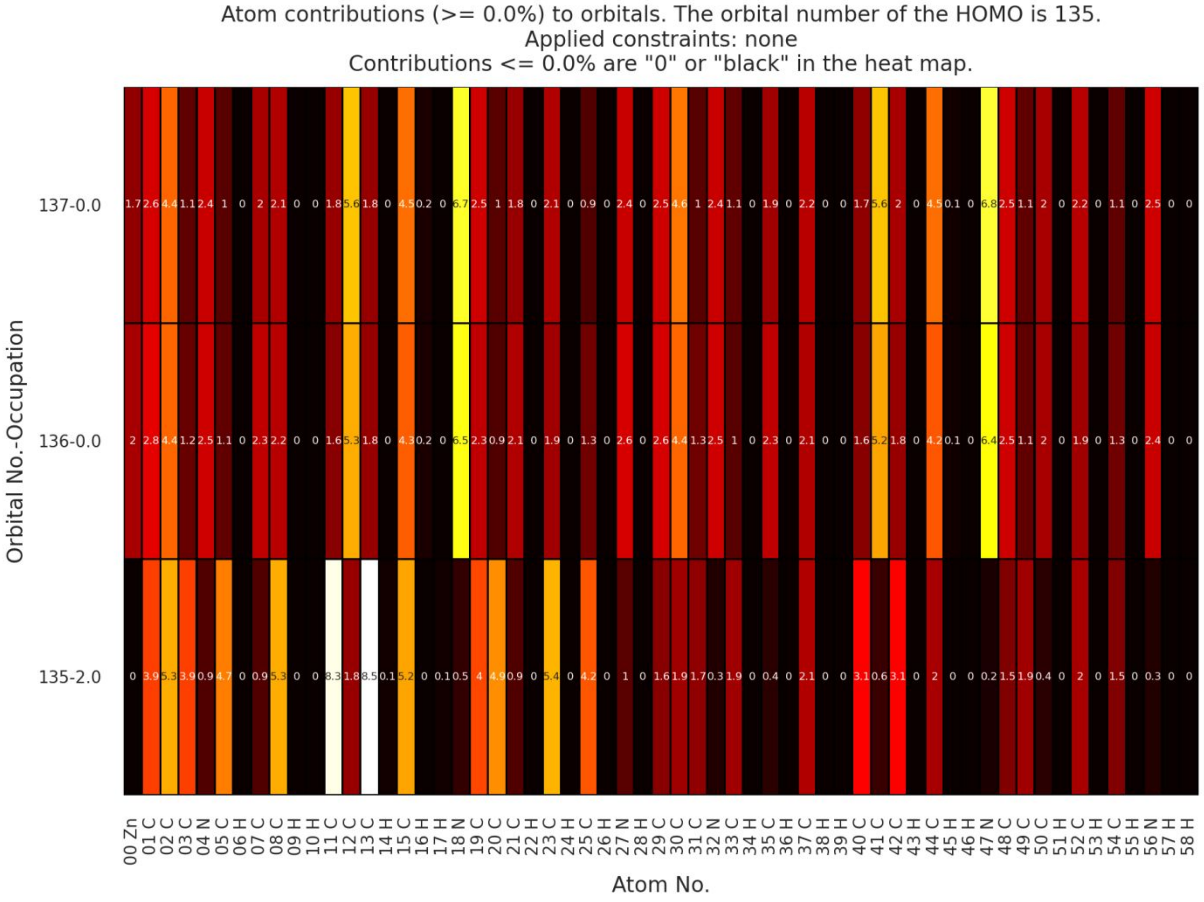

Figure S5. Atomic contributions (%) of the individual Zn, N, C and H atoms to the HOMO (orbital no. 135), LUMO (orbital no. 136) and LUMO+1 (orbital no. 137) of complex $\mathbf{2}_2$•Zn^{2+}. The percentage contributions are reported in the heat map boxes corresponding to each atom.

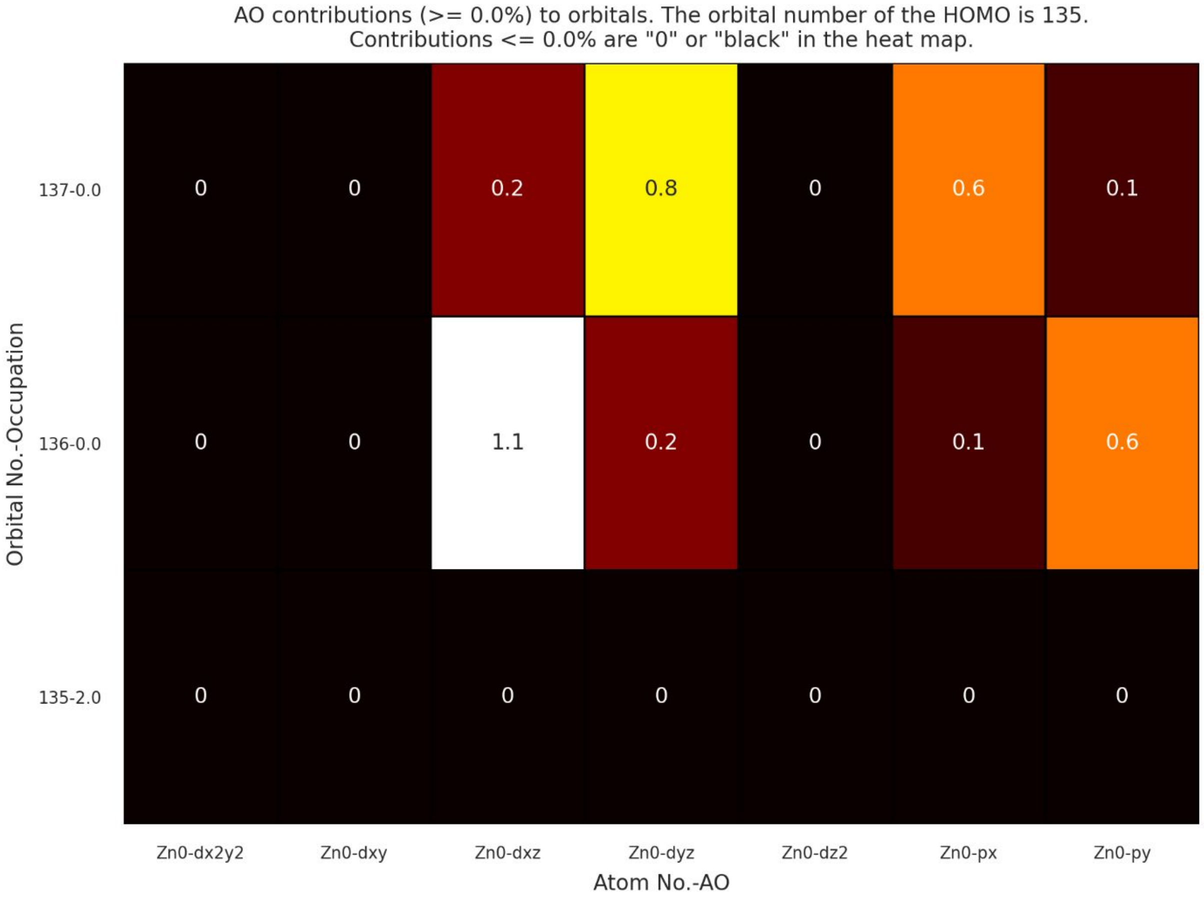

Figure S6. Zinc atomic orbital contributions (%) to the HOMO (orbital no. 135), LUMO (orbital no. 136) and LUMO+1 (orbital no. 137) of complex **2**$_2$•Zn^{2+}. The percentage contributions are reported in the heat map boxes corresponding to each orbital.

2.4 Decomposition of the XANES data into the spectra and fractional concentrations of key components

In order to obtain mechanistic insights into the investigated process, the experimental time resolved Zn K-edge XANES spectra were inserted as columns in a data matrix **D**. In general, a number N of independent components weighed by their fractional concentrations contribute to any given spectrum measured at time t from reaction start, in accordance with the Lambert-Beer law.[8-10] Consequently, **D** was decomposed into the spectra of the key species and in their relative concentration profiles by means of the PyFitit code,[8] which employs to this end an algorithm that belongs to the Multivariate Curve Resolution (MCR) family. Such approach introduces a transformation $N \times N$ matrix **T** in the Singular Value Decomposition (SVD) equation, using the relation $\mathbf{I} = \mathbf{T} \cdot \mathbf{T}^{-1}$, which leads to the following expression:

$$\mathbf{D} = \mathbf{U} \cdot \mathbf{\Sigma} \cdot \mathbf{T} \cdot \mathbf{T}^{-1} \cdot \mathbf{V} + \mathbf{E}$$

where the XANES spectra assigned to the process key species and their associated concentration profiles are $\mathbf{S} = \mathbf{U} \cdot \mathbf{\Sigma} \cdot \mathbf{T}$ and $\mathbf{C} = \mathbf{T}^{-1} \cdot \mathbf{V}$, respectively. In principle, the unknown number of T_{ij} elements of **T** is equal to N^2. By requiring the normalization of all spectral components contained in matrix **S**, the unknown number of the transformation matrix elements is diminished from N^2 to $N^2 - N$. The unknown number of T_{ij} elements may be further reduced by imposing the extracted spectral components to coincide with reference XANES spectra. Since in our case N=2, by constraining the

extracted spectral components to coincide with the normalized XANES spectra of the standards **1**•Zn^{2+} and **2**$_2$•Zn^{2+}, the solutions to the decompositions of the XAS data relative to the reactions involving both chemical fuels were obtained through unique 2 × 2 transformation matrices.

REFERENCES

(1) Neese, F. The ORCA Program System. *WIREs Comput. Mol. Sci.* **2012**, *2*, 73–78.

(2) Becke, A. D. Density-Functional Exchange-Energy Approximation with Correct Asymptotic Behavior. *Phys. Rev. A* **1988**, *38*, 3098–3100.

(3) Stephens, P. J.; Devlin, F. J.; Chabalowski, C. F.; Frisch, M. J. Ab Initio Calculation of Vibrational Absorption and Circular Dichroism Spectra Using Density Functional Force Fields. *J. Phys. Chem.* **1994**, *98*, 11623–11627.

(4) Lee, C.; Yang, W.; Parr, R. G. Development of the Colle-Salvetti Correlation-Energy Formula into a Functional of the Electron Density. *Phys. Rev.* B **1988**, *37*, 785–789.

(5) Grimme, S.; Ehrlich, S.; Goerigk, L. Effect of the Damping Function in Dispersion Corrected Density Functional Theory. *J. Comput. Chem.* **2011**, *32*, 1456–1465.

(6) Weigend, F.; Ahlrichs, R. Balanced Basis Sets of Split Valence, Triple Zeta Valence and Quadruple Zeta Valence Quality for H to Rn: Design and Assessment of Accuracy. *Phys. Chem. Chem. Phys.* **2005**, *7*, 3297–3305.

(7) Pantazis, D. A.; Chen, X.-Y.; Landis, C. R.; Neese, F. All-Electron Scalar Relativistic Basis Sets for Third-Row Transition Metal Atoms. *J. Chem. Theory Comput.* **2008**, *4*, 908–919.

(8) Martini, A.; Guda, S. A.; Guda, A. A.; Smolentsev, G.; Algasov, A.; Usoltsev, O.; Soldatov, M. A; Bugaev, A.; Rusalev, Y.; Lamberti, C.; Soldatov, A. V. PyFitit: The Software for Quantitative Analysis of XANES Spectra Using Machine-Learning Algorithms. *Comput. Phys. Commun.* **2020**, *250*, 107064.

(9) Tavani, F.; Capocasa, G.; Martini, A.; Sessa, F.; Di Stefano, S.; Lanzalunga, O.; D'Angelo, P. Direct Structural and Mechanistic Insights into Fast Bimolecular Chemical Reactions in Solution through a Coupled XAS/UV–Vis Multivariate Statistical Analysis. *Dalt. Trans.* **2021**, *50*, 131–142.

(10) Tavani, F.; Fracchia, M.; Pianta, N.; Ghigna, P.; Quartarone, E.; D'Angelo, P. Multivariate Curve Resolution Analysis of Operando XAS Data for the Investigation of the Lithiation Mechanisms in High Entropy Oxides. *Chem. Phys. Lett.* **2020**, *760*, 137968.
